# Oral delivery of the non-β-blocking R-carvedilol enantiomer for skin cancer chemoprevention in SKH-1 mice

**DOI:** 10.1186/s41120-024-00103-1

**Published:** 2025-01-06

**Authors:** Pabitra K. Sardar, Steven Yeung, Ruby Tow, Jacqueline Luga, Matthew Huang, Ayaz Shahid, Bradley T. Andresen, Ying Huang

**Affiliations:** 1 Department of Biotechnology and Pharmaceutical Sciences, College of Pharmacy, Western University of Health Sciences, Pomona, CA 91766, USA

**Keywords:** Carvedilol, R-carvedilol, Chemoprevention, Skin cancer, SKH-1

## Abstract

**Purpose:**

Skin cancer remains the most prevalent cancer worldwide with its incidence continuously rising. Previous studies have demonstrated the efficacy of the β-blocker carvedilol and its non-β-blocking enantiomer R-carvedilol in mitigating UV-induced skin carcinogenesis through topical application. The current study investigated whether orally administered R-carvedilol could prevent the development of skin cancer in SKH-1 mice.

**Methods:**

Efficacy of orally delivered R-carvedilol was examined in SKH-1 mice exposed to repeated UV radiations for 25 weeks. Pharmacokinetic studies were conduced in mice to evaluate the drug levels in plasma and skin tissues. Pharmacodynamic studies were used to evaluate the effects of oral R-carvedilol and racemic carvedilol on mouse blood pressure.

**Results:**

The findings revealed a statistical difference in tumor incidence between the group receiving R-carvedilol (20 mg/kg) and the UV-only control group (*p* = 0.00860), while lower doses of R-carvedilol (1.5 mg/kg and 5 mg/kg) did not exhibit a significant impact on tumor incidence. While tumor multiplicity varied significantly between groups (*p* = 0.005325), tumor volume analysis showed no statistical difference. Pharmacokinetic studies indicated that R-carvedilol accumulated in a dose-dependent manner within plasma and skin tissues. Notably, at a dosage of 32 mg/kg, oral R-carvedilol did not influence blood pressure, in contrast to carvedilol, highlighting its potential for chemoprevention with minimal cardiovascular side effects.

**Conclusions:**

These data support oral administration of R-carvedilol as a viable strategy for the chemoprevention of skin cancer, given its efficacy and minimal impact on the cardiovascular system. Further studies determining the optimal doses and timing of drug treatment are warranted.

## Introduction

Skin cancer, the prevailing global malignancy, poses an escalating threat to public health, with its incidence rapidly on the rise. The predominant types of skin cancers, namely non-melanoma skin cancers (NMSC), consisting of basal cell carcinoma (BCC) and squamous cell carcinoma (SCC), are witnessing a continuous increase in incidence. These two entities collectively constitute 90% of all malignant skin tumors, and projections indicate a persistent upward trajectory until at least 2040. In the United States alone, the annual diagnosis toll stands at 3.6 million cases for BCC and 1.8 million cases for SCC, as per the most recent data (Tow et al. 2023). Despite heightened awareness and sun protection measures, the incidence of NMSC continues to rise, necessitating more effective preventive strategies (Ciuciulete et al. 2022).

Ultraviolet (UV) radiation is acknowledged as a complete carcinogen, drawing evidence from both human studies and experimental animal research (NTP 2016). The repercussions of excessive exposure to broad-spectrum UV radiation or its constituents (UVA, UVB, and UVC) may lead to the development of NMSC. Sun protection measures, while crucial, have proven insufficient to stem the tide of skin cancer incidence. Consequently, there is a growing imperative to explore chemopreventive interventions that can impede the development of skin cancers. Previous research has shed light on the potential of carvedilol, a non-selective β-blocker with α-blocking and antioxidant activity, in skin cancer chemoprevention (Leiter et al. 2014). Carvedilol holds significance in the management of conditions such as high blood pressure, heart failure, and post-myocardial infarction left ventricular dysfunction. Noteworthy is the fact that carvedilol manifests as two enantiomers, R-carvedilol and S-carvedilol, with distinct pharmacological profiles. While the S-enantiomer primarily exerts β-blocking effects, the R-enantiomer only exhibits α-blocking activity (Stoschitzky et al. 2001). Previous in vivo studies on carvedilol and R-carvedilol used topical formulations (Shamim et al. 2023; Abdullah Shamim et al. 2022; Liang et al. 2021; Huang et al. 2017), which demonstrated that R-carvedilol is as efficient as carvedilol in the prevention of skin cancer when applied topically. Since oral drug delivery has the advantages of being convenient, cost-effective, and usually safe, the option of oral administration of cancer chemopreventive agents should be considered. However, this potential avenue encounters limitations, as carvedilol is highly lipophilic with poor oral bioavailability of 25%–35% due to high first-pass metabolism. Since the effective doses of carvedilol for cancer prevention are in the micromolar range, which is higher than its activity on β-adrenergic receptors (nanomolar range), oral delivery of carvedilol for cancer prevention is a challenge.

The hypothesis of this study is that oral delivery of R-carvedilol (R-CAR) will act as a chemopreventive agent via delaying UV-induced tumor formation without affecting the blood pressure of SKH-1 mice. This hypothesis was addressed first by examining three doses of R-carvedilol, revealing a dose-dependent delay in the onset of skin tumors. Then, drug concentrations in the circulation and skin were examined after oral R-carvedilol administration. Finally, the effects of oral carvedilol and R-carvedilol on the blood pressure of mice were examined. This study represents a crucial step towards determining if oral delivery of R-CAR is a viable chemopreventive agent for skin cancer.

## Materials and methods

### Compounds

Carvedilol was purchased from TCI American (Portland, OR). The optically pure R-CAR was synthesized at Chem-Impex International, Inc. (Wood Dale, IL) and verified by chiral HPLC analysis described in previous studies (Shamim et al. 2023). These compounds were reconstituted in dimethylsulfoxide (DMSO) as a stock solution and stored at –20 °C. Carvedilol and R-CAR were diluted from the DMSO stock in mouse drinking water weekly.

### UV light source

UV lamps emitting UVB (280—320 nm; 54% of total energy), UVA (320–400 nm; 37% of total energy), UVC (100–280 nm; 2.0% of total energy), and Visible light (400–450 nm; 7.0% of total energy) (Catalog numbers #95–0042–08 and #95–0043–13; UVP, Upland, CA) were used to irradiate in vitro and in vivo experiments. Stable power output (mW/cm^2^) was measured using a UVX radiometer (#97–0015–02, UVP) coupled with a sensor with a calibration point of 310 nm (UVX-31, #97–0016–04, UVP). Exposure time was calculated using the following formula: dose (mJ/cm^2^) = exposure time (s) × output intensity (mW/cm^2^). Quality control of the lamps and the exposure time were calculated and monitored before each use of the lamps to account for power output changes.

### UV‑induced murine skin tumorigenesis

All animal studies have been approved by the Western University of Health Sciences Institutional Animal Care and Use Committee (IACUC). Mice used in the present study had access to water and food ad libitum and were housed on a 12-h light/dark cycle in a temperature-controlled facility with 35% humidity. A total of forty 9 ∼ 12-week-old female SKH-1 mice (Charles River) were randomly divided into four groups (*n* = 10): (1) Control: UV only group treated with vehicle; (2, 3, and 4, respectively) R-CAR treatment group exposed to UV and treated with 1.5, 5, and 20 mg/kg/day of R-CAR. The drug treatment started two weeks before the UV exposure began. Three doses were examined for R-CAR. The higher dose (20 mg/kg) was determined based on a previous study examining oral carvedilol in chemical carcinogen 7,12-dimethylbenz(α)anthracene (DMBA) induced skin hyperplasia in mice (Chang et al. 2015). A more clinically relevant lower dose (1.5 mg/kg) was included as using an FDA-recommended formulation for converting equivalent drug dosage between species; 1.5 mg/kg/day in mice is equivalent to 7.5 mg per day in humans (Nair and Jacob 2016). R-Carvedilol was dissolved in DMSO as stock solutions (8.3, 27.8, and 111 mg/mL) and then diluted to the working concentrations into the drinking water. Acidified mouse drinking water (Innovive, M-WB-300A) with a pH ranging between 2.5 to 3.0 is used to prevent the spread of bacterial disease (Whipple et al. 2021). R-CAR, which is considered hydrophobic with a solubility ranging from 5.8–51.9 μg/mL (Hamed et al. 2016), showed increased solubility (> 0.2 mg/mL) in the acidified drinking water. Drug concentrations in the water were adjusted based on average daily water consumption (for detailed dose calculation, refer to previous reports (Wang et al. 2011; Wang et al. 2013; Shahid et al. 2023)). The drinking water was changed weekly. The average water consumption per cage was measured weekly and drug concentrations in the mouse water bottles were adjusted accordingly. Animals under treatment were provided ad libitum access to drug-containing water as the only source of drinking fluid. The mice were irradiated with gradually increasing UV levels three times a week for 25 weeks with an initial dose of 50 mJ/cm2009^2^ that was increased each week by 25 mJ/cm^2^ to 150 mJ/cm^2^, which continued for the duration of the experiment. During the UV exposure, mice roamed freely in acrylic cages on a rotating platform with rotational placement, ensuring consistent and equal dorsal distribution of UV irradiation. Tumors of at least 1 mm in diameter were counted and measured with a Caliper weekly. The tumor volume was calculated according to the formula: (width)^2 × length/2. At 25 weeks, the mice were sacrificed, and tumor tissues were harvested and processed for histological analysis.

### Histological analysis

The skin tumors were fixed in 10% Neutral Buffered Formalin (VWR, Radnor, PA), processed, and embedded in paraffin blocks. 5 µm-thick prepared sections of the paraffin-embedded skin tissues were placed onto positively charged glass slides. The sections were stained with hematoxylin and eosin (H&E). The skin tumors were classified based on reported tumor histology for UV-induced skin tumors in SKH-1 mice (Benavides et al. 2009).

### PK analysis—determination of drug distribution after oral R‑carvedilol dissolved in drinking water

To investigate the distribution of orally administered R-CAR, female SKH-1 mice were treated with R-CAR at a dosage of 20 or 32 mg/kg/day for 1 week. R-CAR was added directly to the acidified drinking water as described above. After the treatment period, mice were sacrificed, and plasma and skin tissue samples were collected. LC/MS/MS was used to quantify the concentration of R-CAR in the samples.

### Sample preparation for LC/MS/MS analysis of R‑carvedilol

A stock solution of R-CAR (200 μg/mL) was prepared by dissolving 2 mg of R-CAR into 10 mL of 50% methanol. The working solutions were prepared by appropriate dilutions of the stock solution with 50% methanol to yield concentrations of 20, 50, 100, 200, 500, 1000, and 2000 ng/mL. An internal standard stock solution was prepared by dissolving 1 mg of propranolol into 10 mL of 50% methanol. The internal standard working solution was prepared by diluting the stock solution 10 times with 50% methanol. The plasma samples for constructing the calibration curve were prepared by spiking blank plasma (90 μL) with 10 μL of corresponding working solutions of R-CAR to yield the following concentrations of 2, 5, 10, 20, 50, 100, and 200 ng/mL and a final concentration of 1 µg/mL internal standard.

Plasma samples were prepared using liquid–liquid extraction. Calibration curve samples were created by using 100 μL of blank plasma, 10 μL of internal standard, and 10 μL of corresponding prepared R-CAR standards in a 1.5 ml centrifuge tube. Experimental samples were processed by using an aliquot of 100 μL of plasma sample spiked with 10 μL of internal standard and 10 μL of 50% methanol in a 1.5 ml centrifuge tube. Both the calibration curve and plasma samples were further processed by extraction with 400 μL of ethyl acetate. These samples were vortexed for at least 2 min and centrifuged at 10,000 rpm for 5 min. The top liquid layer was transferred to a clean 1.5 mL centrifuge tube and dried under nitrogen. The pellet is reconstituted using 100 μL of 50% methanol, and 10 μL was injected into the LC/MS/MS for analysis.

### LC/MS/MS analysis

The LC/MS/MS system consists of an API3200 triple quadruple mass spectrometer (Sciex, Framing-ham, MA), two Shimadzu LC-20AD Prominence HPLC pumps equipped with a SIL-20A Prominence autosampler (Shimadzu, Columbia, MD). All data was analyzed using Analyst 1.5.1 software. The MS conditions were optimized by infusing each analyte (100 ng/mL) at a flow rate of 5 µL/min, which were prepared in 0.1% formic acid:acetonitrile (1:1). The protonated ions of R-CAR (407.138 m/z) and propranolol (260.145 m/z) (internal standard) were detected using the positive ESI mode. The most abundant product ions of 224.2 and 116.058 m/z were selected for R-CAR and propranolol, respectively, and the MS chromatographic conditions were optimized using the Compound Optimization module (FIA mode) built into the Analyst 1.5.1 software. The optimized conditions used were: curtain gas, 25 psi; collision gas, 5 psi; ion spray voltage, 5500 V; temperature, 550 °C; gas 1: nitrogen, 40 psi; gas 2: nitrogen, 20 psi. The MS parameters for R-CAR and propranolol (PRO) are shown below.

**Mass** spectrometry condition to generate transition ions

**Table:** 

MS settings	R‑CAR	PRO
Dwell Time (msec)	300	300
Declustering Potential (DP)	56	41
Entrance Potential (EP)	9	10
Collision Cell Entry Potential (CEP)	18	14
Collision Energy (CE)	42	25
Collision Cell Exit Potential (CXP)	4	4

Chromatography was carried out using an Agilent Zorbax SB-C18 reverse-phase column (2.1 × 150 mm; 5 μm), which was proceeded with a C18 guard column (2.1 × 10 mm, 5 μm) at room temperature. The mobile phases were (A) 2 mM ammonium acetate containing 0.1% formic acid and (B) acetonitrile. A linear gradient elution at a flow rate of 0.3 mL/min was used: beginning with 20% eluent B for 2 min, linearly changed to 80% eluent B for 1 min, remaining constant at 80% eluent B for 4 min, then linearly changing back to 20% eluent B for 1 min and reconditioned at 20% eluent B for 2 min. The total run time was 10 min. The lower limit of quantification for R-CAR in this method is 2 ng/mL with a typical retention time of 5.58 min. Propranolol had a retention time of 5.33 min.

### PD analysis – evaluation of the cardiovascular effects of carvedilol and R‑carvedilol

In order to determine if oral R-CAR has any cardiovascular effects, blood pressure was measured. Twenty-four 12-week-old male SKH-1 mice were divided into three groups (*n* = 8): a negative control without drug treatment, a positive control receiving 32 mg/kg of oral carvedilol in the acidified drinking water, and a group receiving 32 mg/kg of oral R-CAR in the acidified drinking water. Blood pressure was monitored using a CODA 8-Channel High Throughput system (Kent Scientific Corp, Torrington, CT), which utilizes a tail cuff. After a four-week acclimation period where the intra-animal error was less than 5% in the last week, the experiment began. First, blood pressure was measured for two weeks to obtain baseline blood pressure for each mouse. Then, the mice were exposed to the drug-containing acidified water, and their blood pressure was measured for an additional three weeks. Drugs were administered via drinking water as described above, adjusted based on daily water consumption to ensure targeted dosages. After the last blood pressure measurement, the mice were sacrificed, and plasma drug concentrations were analyzed via LC/MS/MS analysis. Due to the variability in plasma CAR and R-CAR levels, the data is presented as the delta for the average of the third week of drug treatment subtracted from the average from the baseline data and compared to the plasma concentration of the respective drug.

### Statistical analysis

All data in the text are described as mean ± SD or SEM, data in histograms are expressed as individual data points with a line representing the group mean, and line graphs as mean ± SD, SEM, or 95% confidence interval (CI); data presentation and error quantification are described in the figure legend. All data were graphed, and curves were generated using beta versions of GraphPad Prism 9.0.0 (La Jolla, CA). NCSS 2019 was used to analyze the data after removing any outliers via ANOVA; Tukey–Kramer post hoc tests were used for parametric data, and Kruskal–Wallis post hoc tests were used for nonparametric data. Tumor formation was graphed using a Kaplan–Meier survival curve showing the incidence of tumors forming in GraphPad Prism and analyzed using a Cox regression for main effects and a Mantel-Cox log-rank test to determine differences in hazard ratios in NCSS 2019. For all statistical analyses, groups were considered statistically different when *p* < 0.05, and the denotation of statistical difference is described in the figure legends.

## Results

### Effects of oral R‑carvedilol on UV‑induced skin carcinogenesis of SKH‑1 mice

**Fig. 1:**
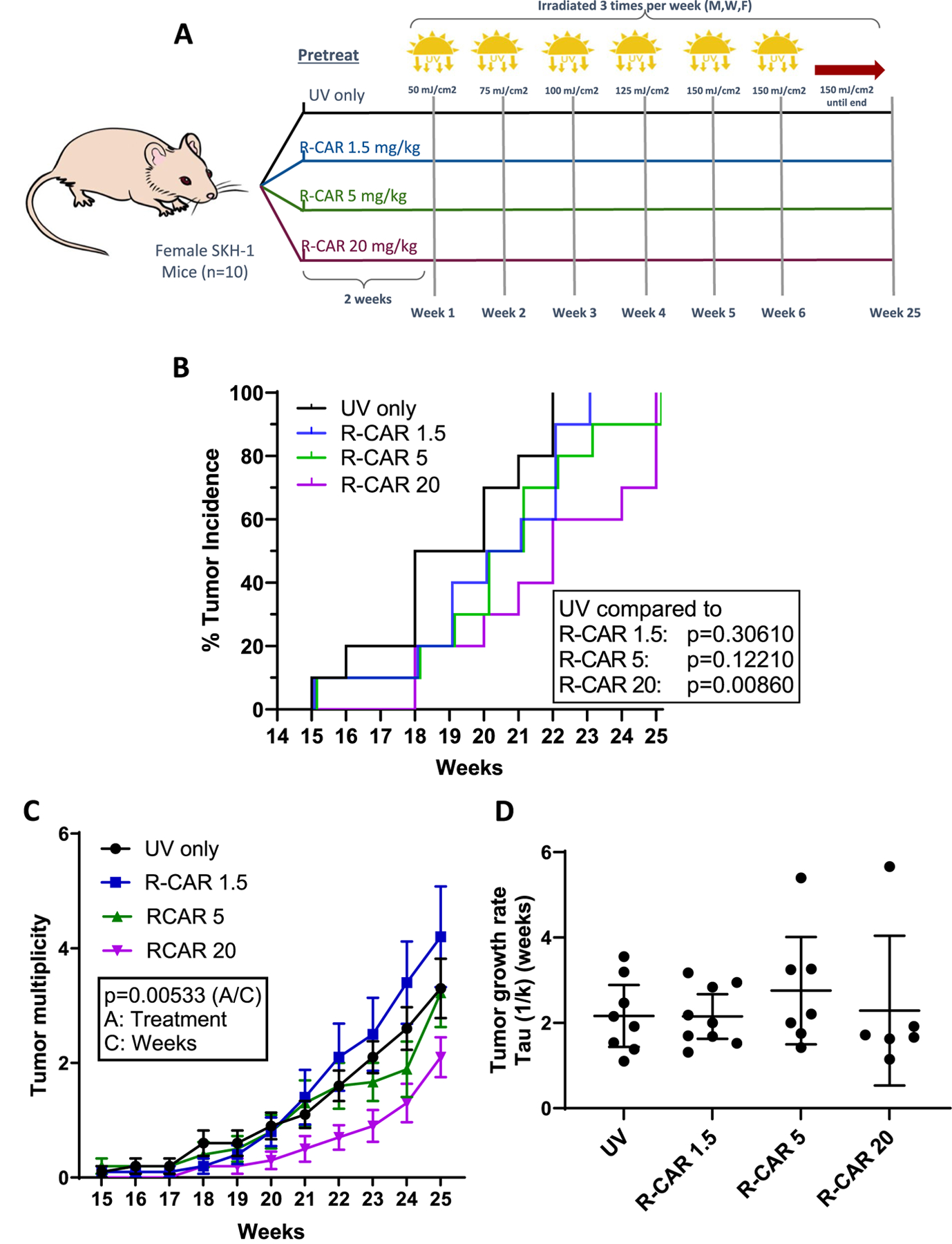
Effects of oral R-carvedilol on UV-induced skin carcinogenesis. **A** Experimental design: 40 female SKH-1 mice divided into four groups (*n* = 10 per group) were administered varying doses of R-CAR in drinking water (1.5, 5, and 20 mg/kg) for two weeks prior to UV irradiation. **B** Tumor incidence over 25 weeks in UV-irradiated mice with or without R-carvedilol treatment; *p*-values displayed are from a Cox regression analysis. **C** Tumor multiplicity: *p*-value is the interaction from an RM-ANOVA. Tukey–Kramer posthoc analysis indicated that only the R-carvedilol 1.5 mg/kg and 20 mg/kg groups were statistically different. **D** Tumor growth rates: *p*-value is from a One-way ANOVA. The sample size for the tumor growth rates varies because not all tumor growth can be fit to an exponential growth curve, and at least three points (weeks) are needed to make the exponential growth curve

We evaluated the tumor incidence, multiplicity, volume, and growth within a 25-week period in a murine model subjected to chronic UV radiation, with and without the administration of R-CAR at various dosages. The groups included a UV-only control and three experimental cohorts receiving 1.5 mg/kg, 5 mg/kg, and 20 mg/kg of R-CAR. The experimental design is shown in Fig. 1A.

The investigation of tumor incidence over the 25 weeks demonstrated that UV-induced tumors in the control group first appeared at week 15, with a median incidence of 18 weeks, and achieved 100% tumor incidence by week 22 (Fig. 1B). The 1.5 mg/kg R-CAR group also showed initial tumor appearance at week 15, with a median incidence of 20 weeks, and achieved 100% incidence by week 23, which was not statistically different from the control (*p* = 0.3061); the Hazard Ratio (HR) of 1.5 mg/kg R-CAR/UV is 0.66 (*p* = 0.3445). The 5 mg/kg R-CAR group also presented initial tumors at week 15, with a median incidence of 20 weeks, and achieved 100% incidence by week 25, which, similar to the 1.5 mg/kg group, was not statistically different than control (*p* = 0.1221); the HR of 5 mg/kg R-CAR/UV is 0.63 (*p* = 0.3037). The 20 mg/kg R-CAR group displayed delayed initial tumor appearance (week 18), with a median incidence of 22 weeks, and achieved 100% incidence by week 25, which was statistically different from the control (*p* = 0.0086); the HR of 20 mg/kg R-CAR/UV is 0.42 (*p* = 0.0476). When comparing the HR of each group (HR was reversed to keep the denominator constant for comparison), the 20 mg/kg R-CAR group was only statistically different from the UV control as previously stated (HR = 2.36, *p* = 0.0476) but not the other R-CAR groups (1.5 mg/kg R-CAR group: HR = 1.94, *p* = 0.1325; 5 mg/kg R-CAR group HR = 1.63, *p* = 0.2698). Although the lower two doses were not statistically different from the UV control, the HR data displays a dose–response relationship.

Tumor multiplicity was counted (Fig. 1C), with the objective of determining if oral R-CAR changes the average tumor number across the 25-week (Fig. 1A). Utilizing a One-way Repeated measures ANOVA to evaluate the data, a statistical difference in tumor volumes across the groups throughout the duration of the study was identified d [F (30, 436) = 1.84, (*p* = 0.005325)]. Post hoc analysis revealed that the difference was primarily between the R-carvedilol 1.5 mg/kg and 20 mg/kg groups. No other groups were statistically different from one another, indicating that R-CAR has a modest effect on tumor multiplicity.

Tumor volume was assessed to determine if oral R-CAR changes the average tumor number across the 25-week experiment (Fig. 1A). Utilizing a One-way Repeated measures ANOVA to evaluate the data, the findings revealed no statistical difference [F (21, 316) = 1.49, (*p* = 0.079972)]; data not shown. Similarly, tumor growth was modeled via fitting tumor volume to an exponential growth curve to determine the growth rates (Fig. 1D). There was no difference in the growth rates [F (3, 29) = 0.5665, (*p* = 0.64203)], and the R-CAR groups appear similar to control. This outcome parallels previous studies with racemic carvedilol (Huang et al. 2017), indicating that the administration of R-CAR, regardless of the dosage, delays tumor formation (Fig. 1B) but does not affect tumor growth once the tumors are formed.

### Effects of 20 mg/kg/day oral R‑carvedilol on histopathology of UV‑induced skin tumors

**Fig. 2:**
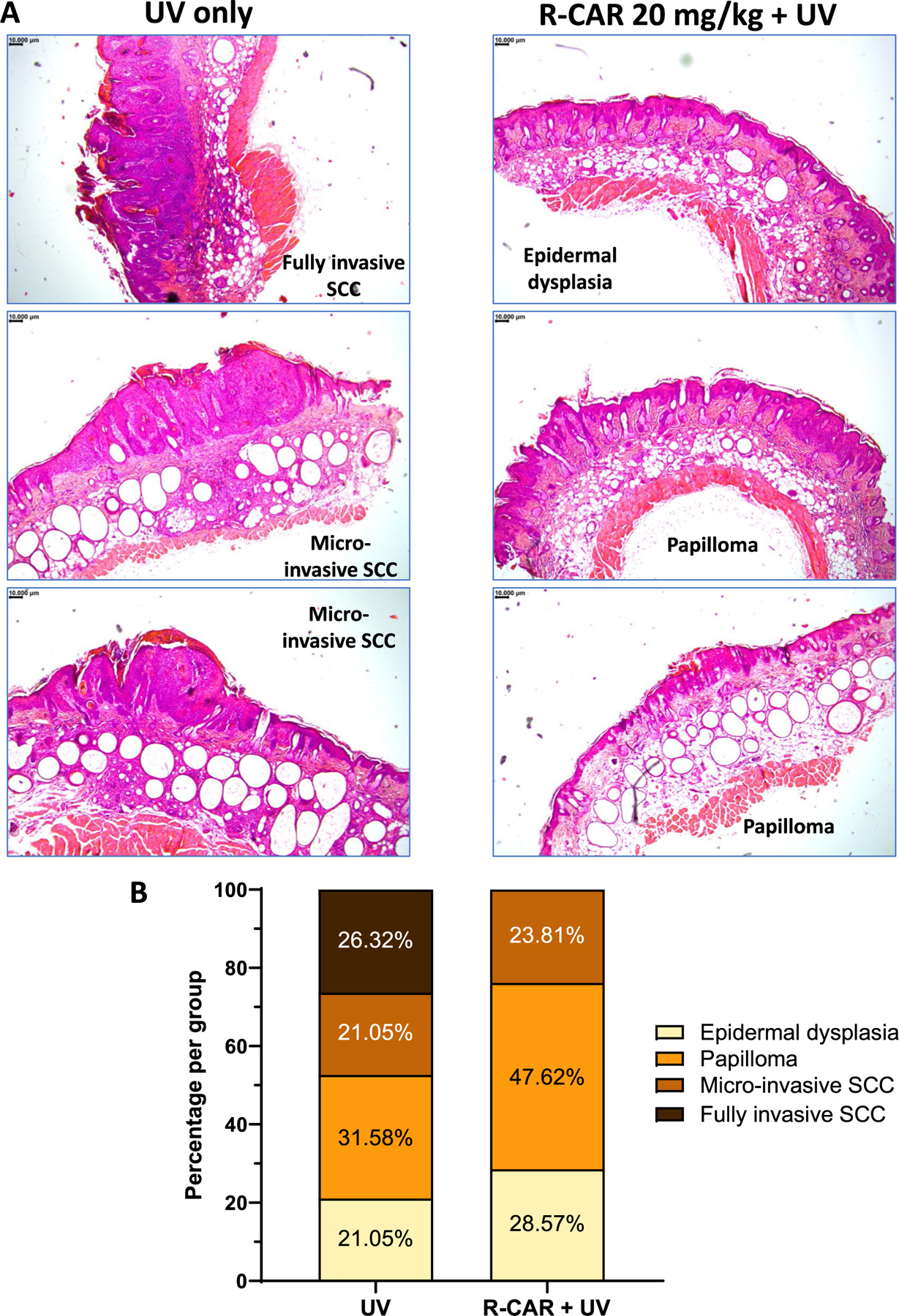
Effects of oral R-carvedilol on histopathology of UV-induced skin tumors. **A** Representative H&E images of skin tumors collected from the control and 20 mg/kg R-carvedilol groups. **B** Characterization of the histology of the two largest skin tumors from each mouse in the control and 20 mg/kg R-carvedilol groups

Histological characteristics of the two largest skin tumors from each mouse in the control and 20 mg/kg R-CAR groups were dissected and fixed in formalin. The skin tumors demonstrated diverse histological characteristics, from premalignant epithelial hyperplasia and papilloma to malignant squamous cell carcinoma (SCC) (Fig. 2). In the UV control group, 47% of the tumors were SCC, while in the 20 mg/kg R-CAR group, 24% were SCC. The UV control group SCC was divided into micro-invasive SCC (44% of SCC) and fully invasive SCC (56% of SSC), whereas there were no fully invasive SCC tumors in the 20 mg/kg R-CAR group. The benign tumors were greater in the 20 mg/kg R-CAR group (76% of all tumors) compared to the UV control (53% of all tumors) (Fig. 2B). These data indicate that oral delivery of 20 mg/kg/day R-CAR treatment reduced the aggressiveness of skin tumors.

### Drug concentrations in plasma and skin after oral R‑carvedilol treatment

The pharmacokinetic profile of R-CAR following oral administration in mice was evaluated to determine the concentration of R-CAR in plasma and skin tissue. R-CAR at two doses, 20 mg/kg and 32 mg/kg, were given to the mice in their drinking water, as described in the Methods, for one week. Detectable levels of R-CAR were observed in both plasma and skin samples post-treatment (Table 1). Importantly, both groups display a large variation in plasma and skin R-CAR levels. It is likely that not all mice consumed the same amount of water. Such variability may impact long-term cancer formation and growth studies. Interestingly, the skin has significantly more R-carvedilol than plasma (*p* = 0.028438). These findings demonstrate that orally administered R-CAR can be concentrated in the skin.

### Oral carvedilol but not R‑carvedilol reduced mouse blood pressure

**Fig. 3:**
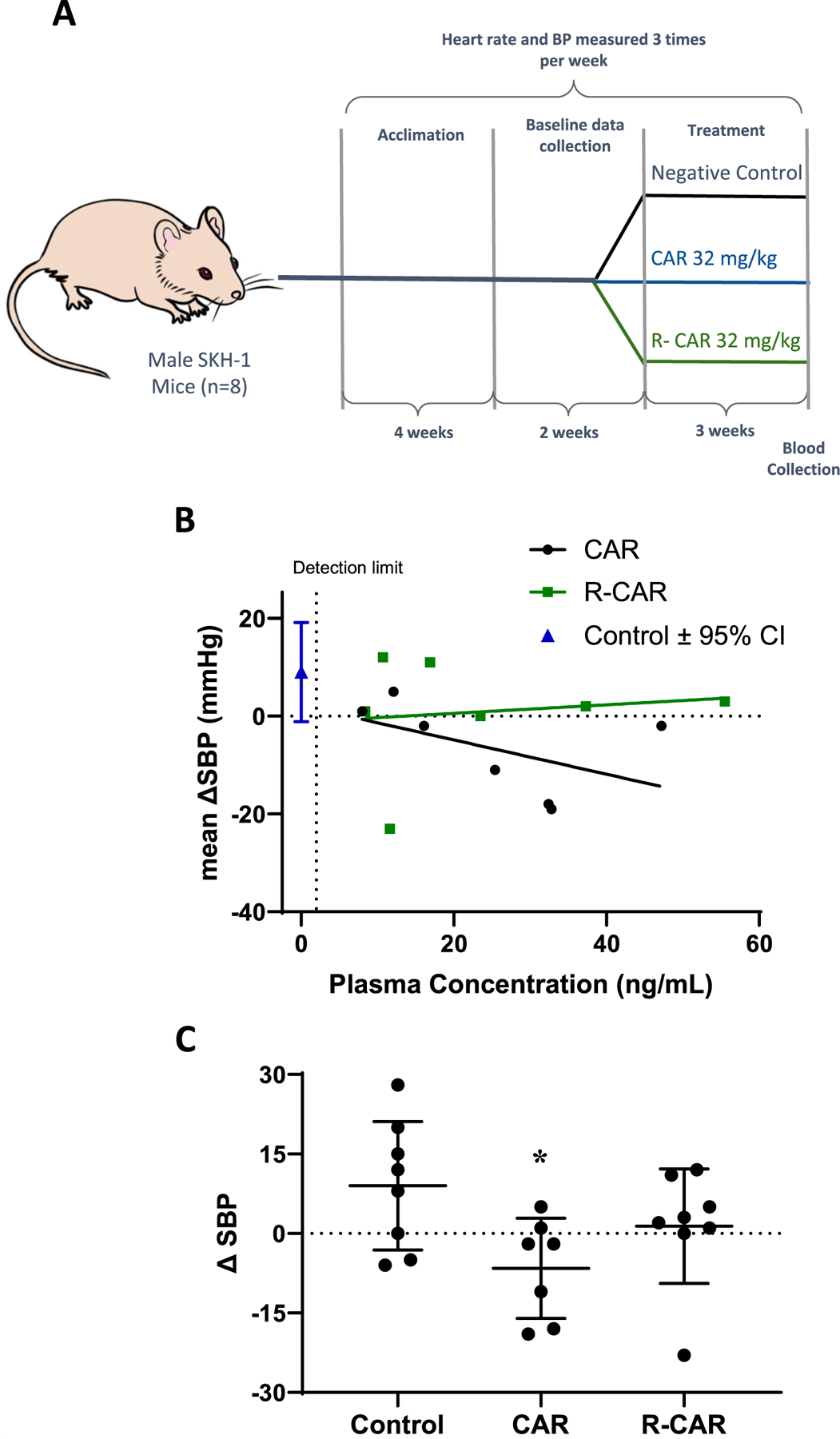
Systolic blood pressure (SBP) of mice treated with carvedilol or R-carvedilol in drinking water. **A** Experimental design to study the effects of oral carvedilol and R-carvedilol on blood pressure. **B** The mean SBP of the baseline data collection was subtracted from the mean SBP of the last week (DSBP) and plotted based on the individual plasma concentration of carvedilol (CAR) and R-CAR. The control group, with no treatment, is shown to depict normal variation. The lines represent the linear correlation between DSBP and plasma concentration of carvedilol/R-CAR. **C** DSBP plotted irrespective of plasma concentration; ANOVA followed by Dunn’s Test of Control indicates that only carvedilol reduced systolic blood pressure (*p* < 0.05)

The effects of oral administration of carvedilol (CAR) and R-CAR on mouse blood pressure were assessed to compare the systolic blood pressure (SBP) changes among groups treated with vehicle (negative control), CAR (32 mg/kg/day), and R-CAR (32/mg/kg/day) over 28 days (Fig. 3A). Utilizing drinking water to deliver the drugs resulted in a similar mean plasma concentration (and 95% confidence interval (CI)) of 32.29 (16.71 to 47.74) and 25.13 ng/mL (7.81 to 42.46) for CAR and R-CAR, respectively. As previously described, the variation within each group is high, which impacted the mean effect on SBP; therefore, the change in blood pressure was compared to the plasma concentration of CAR and R-CAR, respectively (Fig. 3B). Due to poor readings and an animal that passed away, only seven mice were in the final analysis of CAR and R-CAR groups. The control DSBP had a mean of 9 mmHg with a 95% CI of −1 to 19 mmHg. The DSBP in the CAR group negatively correlated with plasma concentrations of CAR (*r* = −0.6847, *p* = 0.0500), whereas the R-CAR group had a slight, non-statistically different, positive correlation with plasma concentrations of R-CAR (*r* = 0.03571, *p* = 0.4817). Depicted as a histogram (Fig. 3C), an ANOVA provided *p* = 0.0648, above the traditional statistical cutoff; however, Dunn’s multiple comparisons to control revealed that there is only a difference in the CAR group (*p* = 0.0421), not R-CAR (*p* = 0.8766). Although the sample size proved small, the outcome is consistent with CAR lowering SBP and that R-CAR cannot lower SBP, as shown previously with a 1.6 mg/kg dose of R-CAR in mice (Zhang et al. 2015).

## Discussion

The non-*β*-blocking R-carvedilol enantiomer has demonstrated promising potential in skin cancer chemoprevention, with topical application offering a similar efficacy to that of carvedilol (Liang et al. 2021). One significance of the present study lies in the fact that oral delivery of 20 mg/kg R-carvedilol reduces tumor incidence (Fig. 1) at a mean plasma concentration similar to what is achievable in humans (Kim et al. 2015), and the drug R-carvedilol is concentrated in the skin (Table 1). The second significance is to confirm that R-carvedilol lacks β-blocking properties, which are characteristic of the β-blocker carvedilol and associated with the clinical effects of carvedilol, specifically reduction in heart rate and blood pressure (Fig. 3). The absence of such β-blocking effects with R-carvedilol may represent a significant advantage as cardio-depressive effects are not ideal for a chemopreventive agent that healthy individuals would take. Thus, oral delivery of R-carvedilol could provide a safe long-term chemopreventive therapy for skin cancer as an oral agent already FDA-approved for clinical use.

In this study, orally delivered 20 mg/kg R-carvedilol decreases tumor incidence compared to UV control alone (Fig. 1B). Notably, this decrease is seen when plasma R-carvedilol levels from an independent group of animals varied significantly (Table 1). The delivery of R-carvedilol in drinking water is convenient for a long-term study with a drug known to have poor bioavailability (Wang et al. 2013); however, it leads to considerable variation in plasma concentrations. Due to the study design, it is not feasible to determine the plasma concentrations in the chronic UV-exposed mice without causing undue stress, which is a known contributor to cancer development (Saul et al. 2005). The variations in plasma concentrations likely influenced the tumor incidence data. The presumed variation may explain why doses lower than 20 mg/kg were not effective, suggesting a more controlled dosing mechanism will be required to determine the minimal effective dose. However, the presumed variation may model patients who do not regularly take their medication and thus mimic the real-world use of oral R-CAR as a chemopreventive agent.

Unlike previous studies with topical carvedilol (Huang et al. 2017), oral delivery of R-CAR failed to significantly reduce the tumor multiplicity compared to the UV control (Fig. 1C). Due to the aforementioned high variation in R-CAR plasma levels, the experiment is underpowered to quantify differences in tumor multiplicity. It is expected that a drug that delays tumors would display lower tumor multiplicity, which is observed with topical carvedilol (Huang et al. 2017). Future studies should increase the sample size based on this data set to determine if R-CAR reduces tumor multiplicity. Alternatively, an alternative dosing mechanism reducing the plasma variability could be used to determine if R-CAR reduces tumor multiplicity.

To date, all studies conducted with carvedilol (Shamim et al. 2023; Abdullah Shamim et al. 2022; Huang et al. 2017; Chang et al. 2015) and this study with R-CAR demonstrate that carvedilol/R-CAR does not affect tumor growth (Fig. 1D). Although tumor formation is delayed, the 20 mg/kg R-CAR group first displayed tumors when half the UV control mice displayed tumors; once the tumors formed, they grew at the same rate. The difference in age of the tumors between the 20 mg/kg R-CAR and UV groups likely explains the difference observed in the severity of the tumors (Fig. 2). Previous studies utilizing carvedilol also report a decrease in tumor severity and also display similar changes in tumor incidence (Huang et al. 2017). Further studies should be conducted where age-matched tumors are extracted from the animals to determine if carvedilol or R-CAR displays any difference in tumor progression. Regardless of time as a variable, it is evident that the mechanism of carvedilol and R-CAR prevention of tumors is distinct from mechanisms that are pharmacologically targeted to combat tumor growth. Future studies should be designed to elucidate the chemopreventive mechanism(s) to understand how R-CAR delays UV-induced tumor formation.

It is critical to acknowledge the limitations of repurposing R-CAR for cancer prevention. Foremost, current FDA regulations require proof of efficacy for a drug to be approved for an indication. For cancer prevention, and for that matter, any chronic disease, such a clinical trial will be difficult and costly due to the time required for such a human trial. Retrospective clinical studies support that oral racemic carvedilol has cancer-preventative properties (Lin et al. 2015); however, no prospective trial has been attempted. Secondly, although our findings underscore that convenient oral administration is effective, the bioavailability of carvedilol is low (Kim et al. 2015; Morgan 1994). Oral administration of R-CAR may be effective for skin cancer due to the accumulation of R-CAR within the skin (Table 1). However, it is expected that oral R-CAR could be used to prevent other cancers (Shahid et al. 2023; Gillis et al. 2021). Topical R-carvedilol does reduce skin cancer, but such a formulation, although plausible for skin cancer, would likely not help prevent other cancers identified to be preventable by oral carvedilol. Thirdly, although R-carvedilol is not a β-blocker, it still exhibits activity of an α1-blocker (Giannattasio et al. 1992; Nichols et al. 1989; Bartsch et al. 1990). Most adverse reactions associated with an α-blocker are caused by dilation of the blood vessels, including hypotension, bradycardia, and arrhythmias. In addition, R-carvedilol, similar to S-carvedilol, has demonstrated activity to reduce the opening frequency of the ryanodine receptors which are calcium channels located in the sarcoplasmic/endoplasmic reticulum membrane (Zhou et al. 2011). The ryanodine receptors play an important role for the release of Ca^2+^ from sarcoplasmic/endoplasmic reticulum during excitation–contraction coupling in cardiac and skeletal muscles (Mochizuki et al. 2007). Disturbance of the calcium signaling may cause adverse reactions. Thus, the potential side effects of oral R-carvedilol should be further examined in animal models before regulatory approval of this agent for clinical trials. In addition, ongoing studies in our group are focused on understanding the mechanisms for R-carvedilol’s skin cancer preventive activity. Previous studies have shown that carvedilol and R-carvedilol inhibit UV-induced DNA damage and the production of inflammation biomarkers such as prostaglandin E2 (PGE2) and IL-6, and also reduce UV-induced signaling pathways including AP-1, NF-κB, and MAPK (Liang et al. 2021; Huang et al. 2017; Chen et al. 2020; Cleveland et al. 2018). However, the molecular target(s) that are upstream of these genes/pathways remain to be identified.

This study offers foundational data essential for advancing research aimed at refining the delivery and effectiveness of R-CAR for skin cancer prevention. Future investigations may concentrate on developing new formulations or delivery mechanisms to enhance the oral bioavailability of R-CAR, thus either bolstering its preventive attributes against skin cancer or allowing for a reduced dosage to have substantial effects. Additionally, identifying the mechanism of action may allow for novel therapies that may be as, or more, effective than R-CAR while avoiding the bioavailability issue of carvedilol.

## Conclusions

In conclusion, this study investigated the potential of oral R-CAR for chemoprevention against UV-induced skin carcinogenesis in SKH-1 mice. The results indicated that oral dosing of 20 mg/kg R-CAR demonstrated a statistically significant reduction in tumor incidence compared to the UV-only control group but lacked any effect on tumor growth once tumors were formed. Notably, lower doses of R-CAR, 1.5 and 5 mg/kg, failed to delay UV-induced tumors, suggesting further doses between 20 mg/kg and 5 mg/kg, as well as higher doses than 20 mg/kg, should be explored to understand what dose is most appropriate. Concomitant studies that determine the plasma concentration of R-CAR are essential to determine the effective plasma concentration of R-CAR required to inhibit carcinogenesis. Further studies should be designed to determine if the skin serves as a reservoir of R-CAR. As expected, in contrast to carvedilol, R-CAR did not decrease systolic blood pressure, high-lighting R-CAR as a good candidate for chemoprevention as it lacks cardiovascular effects. Although the plasma concentration varied greatly, orally delivered 20 mg/kg R-CAR is sufficient to delay tumor formation.

## Figures and Tables

**Table 1:** Drug concentrations in plasma and skin tissue after giving R-carvedilol 20 mg/kg and R-carvedilol 32 mg/kg in mouse drinking water for one week

R-CAR dose	Plasma Concentration (ng/g)		Skin Concentration (ng/g)	
	Mean	SD	Mean	SD
20 mg/kg	22.36	33.75	110.51	113.48
32 mg/kg	21.31	23.33	202.20	141.54

R-CAR was measured in the plasma and skin of each mouse. RM-ANOVA displayed no difference in the two doses (p = 0.244890), a difference between plasma and skin (p = 0.028438), but no interaction (p = 0.417656)

## Data Availability

All data generated or analysed during this study are included in this published article.
